# Anti-Apoptotic Effects of Lentiviral Vector Transduction Promote Increased Rituximab Tolerance in Cancerous B-Cells

**DOI:** 10.1371/journal.pone.0153069

**Published:** 2016-04-05

**Authors:** Benyamin Ranjbar, Louise Bechmann Krogh, Maria Bach Laursen, Maria Nascimento Primo, Sara Correia Marques, Karen Dybkær, Jacob Giehm Mikkelsen

**Affiliations:** 1 Department of Biomedicine, Aarhus University, Aarhus, Denmark; 2 Department of Hematology, Aalborg University Hospital, Aalborg, Denmark; 3 Department of Clinical Medicine, Aarhus University, Aarhus, Denmark; Cornell University, UNITED STATES

## Abstract

Diffuse large B-cell lymphoma (DLBCL) is characterized by great genetic and clinical heterogeneity which complicates prognostic prediction and influences treatment efficacy. The most common regimen, R-CHOP, consists of a combination of anthracycline- and immuno-based drugs including Rituximab. It remains elusive how and to which extent genetic variability impacts the response and potential tolerance to R-CHOP. Hence, an improved understanding of mechanisms leading to drug tolerance in B-cells is crucial, and modelling by genetic intervention directly in B-cells is fundamental in such investigations. Lentivirus-based gene vectors are widely used gene vehicles, which in B-cells are an attractive alternative to potentially toxic transfection-based methodologies. Here, we investigate the use of VSV-G-pseudotyped lentiviral vectors in B-cells for exploring the impact of microRNAs on tolerance to Rituximab. Notably, we find that robust lentiviral transduction of cancerous B-cell lines markedly and specifically enhances the resistance of transduced germinal center B-cells (GCBs) to Rituximab. Although Rituximab works partially through complement-mediated cell lysis, increased tolerance is not achieved through effects of lentiviral transduction on cell death mediated by complement. Rather, reduced levels of PARP1 and persistent high levels of CD43 in Rituximab-treated GCBs demonstrate anti-apoptotic effects of lentiviral transduction that may interfere with the outcome and interpretation of Rituximab tolerance studies. Our findings stress that caution should be exercised exploiting lentiviral vectors in studies of tolerance to therapeutics in DLBCL. Importantly, however, we demonstrate the feasibility of using the lentiviral gene delivery platform in studies addressing the impact of specific microRNAs on Rituximab responsiveness.

## Introduction

Diffuse large B-cell lymphoma (DLBCL) is the most common type of non-Hodgkin Lymphoma in adults with a 5-year overall survival rate of 60%, illustrating that some patients are either unresponsive to the current treatment or develop drug resistance during treatment. DLBCL is both clinically and molecularly a heterogeneous disease. The largest subtype is defined as DLBCL, not otherwise specified (DLBCL, NOS), which by gene-expression profiling can be divided into the following molecular subclasses: (i) germinal center B-cell (GCB) DLBCL and (ii) activated B-cell (ABC) DLBCL, [1–3]. The two molecular subclasses GCB and ABC differ in signaling pathway defects, genetic abnormalities, and pathogenesis [1,4–6]. Survival outcomes also differ, as GCB patients have a higher 5-year survival rate of 69–79% compared to 52–53% for those with ABC DLBCL when treated with a standard immuno- and anthracycline-based multidrug chemotherapy regimen, known as R-CHOP, consisting of Rituximab (R), cyclophosphamide (C), doxorubicin (H), vincristine (O), and prednisone (P) [7]. Moreover, approximately one-third of DLBCL patients develop relapsed/refractory disease. Thus, discovery of novel biological markers and therapeutic agents as well as strategies to overcome drug resistance remain key challenges for offering improved treatment to DLBCL patients.

Rituximab is the first FDA-approved antibody to be used in treatment of DLBCL. Rituximab targets CD20 molecules on the surface of pre-B-cells and more differentiated B-cell stages [8]. CD20 is a differentiation-specific cell surface antigen, which is present on the B-cell surface from early stages of B-cell development to post-germinal maturation stages, but not on the surface of mature plasma cells [8]. The molecule is involved in the activation and proliferation of B-cells and is thought to be part of a cell surface complex involved in calcium transport although the ligand for CD20 remains unidentified [8]. Different mechanisms of action have been described for Rituximab, including Complement-Dependent cell Cytotoxicity (CDC), Antibody-Dependent Cell-mediated Cytotoxicity (ADCC), and direct induction of cell death by apoptosis [8,9]. However, despite the benefits of Rituximab, drug resistance remains a challenge for efficient and prolonged therapy. Several mechanisms leading to Rituximab tolerance have been described; These include down-regulation of CD20 expression [10–12], down-regulation of apoptosis-involved proteins such as Bak and Bax [13], and inhibition of P38 MAPK activity [14], as recently reviewed by Pérez-Callejo and colleagues [15]. Additionally, microRNAs (miRNAs) are believed to contribute to the drug response and potential resistance through their capacity to modulate the expression of proteins of key signal transduction pathways [16–18].

MiRNAs are small (around 20 nucleotides) non-coding RNAs that post-transcriptionally regulate gene expression via the RNA interference pathway [19]. These RNA molecules, expressed from RNApolII- or RNApolIII-transcribed genes in the genome, are processed both in the nucleus and, after nuclear export, in the cytoplasm. As part of the RNA-induced silencing complex (RISC), mature miRNAs anneal to recognition sites in target mRNAs and mediate mRNA degradation or translational suppression [20,21]. The interaction between miRNA and mRNA is based on base pairing, but complete match between the two molecules is not required for miRNA function. Hence, a single miRNA has the potential to target and regulate many different mRNAs, whereas a specific mRNA can be targeted by a set of different miRNAs. This creates a network of gene regulatory interactions and suggests that miRNAs play a buffering role in gene regulation with some potential redundancy between miRNAs. Considering these activities, miRNAs are critical players in many cellular and developmental pathways and assist in regulating basic cellular properties including differentiation, proliferation, apoptosis, and homeostasis. It is well-known that miRNA regulation influences cancer development and progression [22], and miRNAs may serve either as tumor suppressors or oncogenes based on their target gene(s) assisting in suppression and promotion, respectively, of cancer growth and progression [23–26].

Global miRNA expression profiling studies have revealed distinct miRNA signatures for different DLBCL subgroups, suggesting that B-cell cancers can be classified based on miRNA expression profiles [27]. Given such differences in miRNA signatures, specific subsets of miRNAs can be identified as potential prognostic and predictive biomarkers for disease progression and treatment, respectively. As an example, high expression of miR-155 was found in a clinical dataset to be associated with failure of R-CHOP treatment [27]. A recent systematic survey of studies focusing on miRNA expression in DLBCL identified a total of 30 miRNAs associated with patient outcome [28]. Notably, only a relatively small number of miRNAs have been identified in several independent studies, supporting the notion that discrepancies may exist due to the extreme regulatory complexity and the existence of largely unidentified networks of cooperating miRNAs. Furthermore, it has been shown that miR-224 can affect the Rituximab efficiency via regulation of the target gene encoding CD59, indicating plausible roles of miRNAs in the development of resistance to Rituximab [29]. A recent study suggested that miR-199a and miR-497 cause increased sensitivity to Rituximab and other chemotherapeutics, contributing to improved overall survival in DLBCL [30].

Global analyses of clinical samples using array-based technologies or next-generation sequencing are powerful approaches, which have led to the identification of molecular predictors, including miRNAs, of DLBCL survival and prognosis [31,32]. It is essential, however, to model miRNA function in B-cells and establish a platform for studying miRNA networks and their importance for the drug response and development of potential drug tolerance. Molecular manipulation in B-cells is complicated by the fact that these cells are difficult to transfect with plasmid DNA and synthesized or *in vitro*-transcribed RNA and that transfection by chemical treatment or electroporation can be accompanied by substantial toxicity and cell death. Virus-based gene transfer, however, represents an alternative strategy for modelling miRNA function in B-cells. In this study, we investigated the use of vesicular stomatitis virus glycoprotein G (VSV-G)-pseudotyped lentiviral vectors in B-cells for studying the impact of miRNAs on the sensitivity of cancerous B-cells to Rituximab. Notably, we show that transduction of GCB-type B-cells with standard lentiviral vectors leads to increased tolerance to Rituximab and that anti-apoptotic factors are induced by treating the cells with lentiviral vectors. Despite these effects of lentiviral vector transduction of B-cells, we demonstrate the feasibility of this gene delivery platform in studies addressing the importance of specific miRNAs in relation to Rituximab responsiveness.

## Materials and Methods

### Cell lines

Six DLBCL cell lines were used for dose-response assays. NU-DHL-1 and SU-DHL-5 were purchased from DSMZ (German Collection of Microorganisms and Cell Cultures). FARAGE [33], OCI-Ly-7 [34], RIVA [35], and SU-DHL-8 [36] were kindly provided by Dr. Jose A. Martinez-Climent (Molecular Oncology Laboratory, University of Navarra, Pamplona, Spain). All cells were cultured at 37°C with 5% CO_2_. DLBCL lines were seeded in RPMI1640 (Lonza, Basel, Switzerland) supplemented with 10% FBS, 1% Glutamine and 1% Penicillin/streptomycin. HEK-293T cells were seeded in DMEM (Lonza, Basel, Switzerland) containing 5% FBS and 1% Penicillin/streptomycin. The identity of the cell lines was verified by DNA barcoding, as previously described [37].

### Vector construction

A standard lentiviral miRNA expression vector (pLV/miRCS-PE), carrying a miRNA cloning site (miRCS) for insertion of PCR-amplified miRNA sequences, was created by cloning the U1 expression cassette containing an internal cloning site (U1-MCS-U1terminator) into the lentiviral vector plasmid pLV/PGK-eGFP [38], which contains the PGK-eGFP (PE) expression cassette. To allow insertion of NotI-digested fragments downstream of the U1 promoter, a Bsp120I restriction site was introduced between the U1 promotor and the U1 terminator. Genomic DNA sequences encoding the various studied miRNAs were PCR-amplified from human genomic DNA isolated from HeLa cells and cloned into Bsp120I-digested pLV/miRCS-PE. Studied miRNAs and the primers used for PCR amplification are provided in S1 Table.

### Analysis of miRNA function

For functional studies of the cloned miRNA variants, individual target sequences were fused to the Rluc reporter gene by insertion of annealed oligonucleotides containing the target sequence into NotI/XhoI-digested psiCHECK-2 vector (Promega, Madison, WI, USA), as described previously [39]. Functionality of cloned miRNAs was checked in HEK-293T cells co-transfected with the psiCHECK-2-derived plasmid and pLV/miRCS-PE-derived plasmid encoding the relevant miRNA using the Dual luciferase assay (Promega, Madison, WI, USA) according to manufacturer’s protocol.

### Lentivirus production and transduction

To produce lentiviral vectors, HEK-293T cells were plated in 10 ml DMEM at a density of 4 × 10^6^ per 10-cm dish. On the next day, cells were transfected with lentiviral packaging plasmids (13.0 μg pIntg, 3.75 μg pMD2G, 3.0 μg pRSV-Rev, and 13.0 μg pLV/miR[number]-PE). Medium was changed after 24 hours. 48 hours after transfection, the supernatant was collected and ultra-centrifuged through a sucrose cushion. The virus yield was assessed using a p24 ELISA kit (XpressBio, Frederick, MD, USA), and the amount of virus for different vector preparations was normalized based on the level of p24 protein. Cancerous B-cells were seeded at a density of 3 × 10^4^/ml in 2 ml RPMI1640. Cells were transduced on the following day using equal amounts of virus based on p24 measurements. The transduction efficacy was measured using flow cytometry (LSRFortessa, BD Bioscience, San Diego, CA, USA).

### Rituximab cytotoxicity assay

To investigate the effects of the various miRNAs on the sensitivity to treatment with Rituximab, transduced cells were collected, washed, and seeded at 3 × 10^5^/ml in 0.8 ml RPMI1640, 72 hours after transduction. Cells were treated the next day with Rituximab at a dose corresponding to the GI50 of Rituximab or the same volume of sodium chloride buffer. A volume of 200 μl pooled Human AB Serum (HS) (Innovative Research, Novy, MI, USA) was added to the plates. Forty-eight hours after drug treatment, cells were counted directly by the trypan Blue dye (as viability marker) exclusion method using the Neubauer chamber (0.0025 mm^2^) and stained in parallel with BrdU (BD Bioscience, San Diego, CA, USA) to measure the proliferation rate. For some experiments, the complement proteins were inactivated in human serum (inHS) by heating at 56°C for 30 minutes.

### Flow cytometry

Cells were collected, washed, and stained for 30 minutes with fixable near IR-dead cell viability marker (Life Technologies, Carlsbad, CA, USA) according to the manufacturer’s protocol. The cells were then washed and fixed in 4% paraformaldehyde for 15 minutes and analyzed for viability and GFP expression. For CD43 and CD20 analysis, cells were collected, washed, and stained with APC-conjugated anti-CD43 (BD Bioscience, San Diego, CA, USA) and APC-conjugated anti-CD20 (BD Bioscience, San Diego, CA, USA) separately for 30 minutes at 4°C in the dark and then fixed 15 minutes with 4% paraformaldehyde. To measure the rate of apoptosis, cells were collected, washed, permeabilized, and stained with PE-conjugated anti-Cleaved PARP1 (BD Bioscience, San Diego, CA, USA) according to the manufacturer’s protocol. To measure the proliferation rate, cells were incubated with BrdU for 55 minutes and then collected, washed twice, and permeabilized and stained with APC-conjugated anti-BrdU (BD Bioscience, San Diego, CA, USA) according to the manufacturer’s protocol. Quantification of GFP, viability, CD43 and CD20 expression, cleaved PARP1, and BrdU-positive cells was assessed with a flow cytometer (LSRFortessa, BD Bioscience, San Diego, CA, USA) and the data analyzed using FlowJo_V10 software.

### Quantitation of mRNA levels by qPCR

Cells were harvested, washed, and lysed 48 hours after Rituximab treatment. Total RNA was purified using mirVana miRNA Isolation Kit (Life Technologies, Carlsbad, CA, USA) according to the manufacturer’s protocol. The expression of CD43 was measured by primers and probe (Applied Biosystems, Waltham, MA, USA) according to the manufacturer’s protocol using Light Cycler 480 (Roche, Basel, Switzerland). The level of expression was normalized to the expression of RPLP0.

### Statistical analysis

Student’s t-tests (two tailed) paired and unpaired were performed. All experiments including drug assays and gene and protein expression analysis were performed three times in biological triplicates.

## Results

### Efficient lentiviral transduction of B-cells of GCB- and ABC-like origin

**Fig 1:**
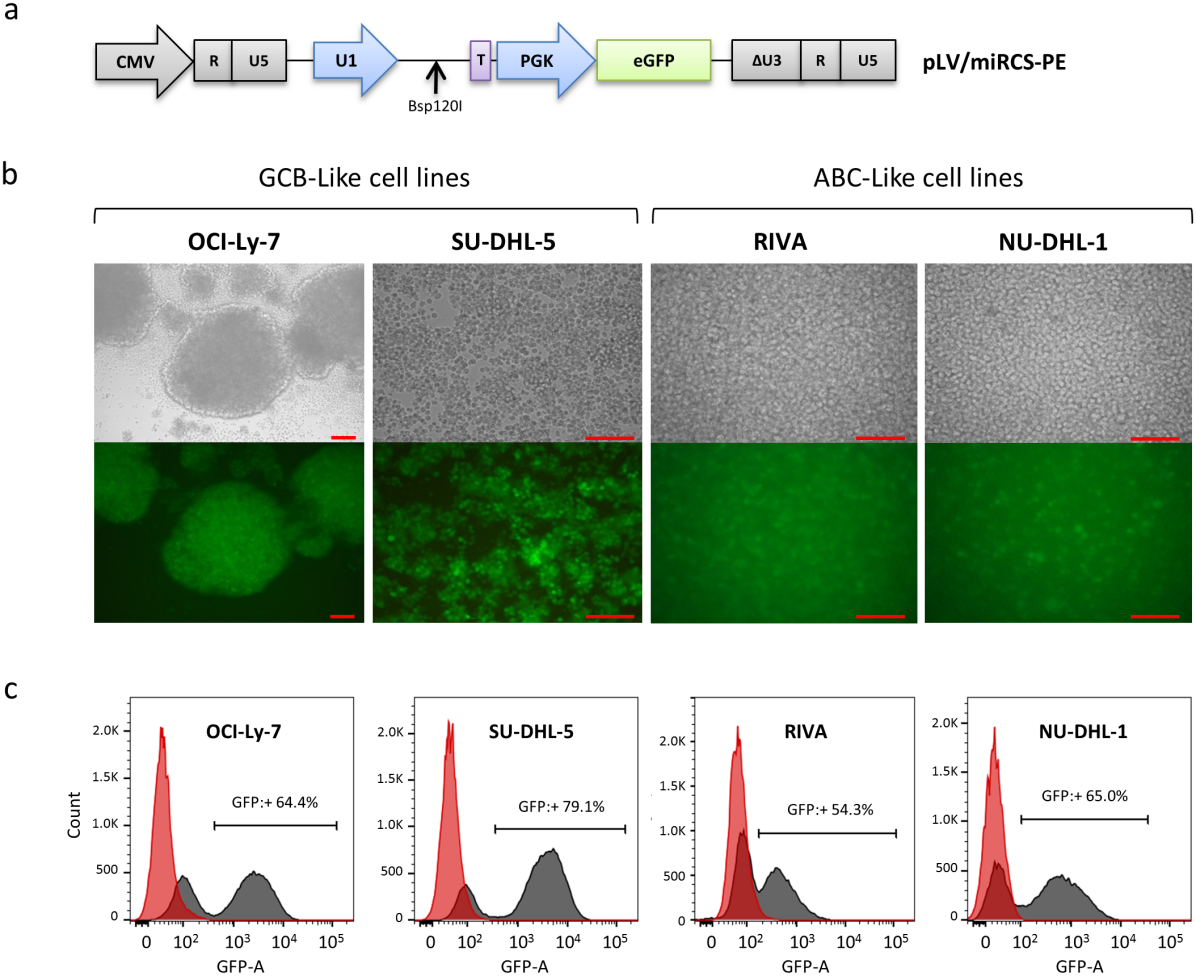
Robust lentiviral transduction of cancerous B-cell lines. **(a)** A lentiviral vector, pLV/miR-PE was constructed to simultaneously express a specific miRNA (from a U1 promoter) and eGFP (from a PGK promoter). The purple box (marked ‘T’) indicates the U1 terminator sequence that terminates transcription of miRNAs inserted into the BSp120I site. Light grey boxes illustrate elements (R, U5, and ΔU3) of the long terminal repeats of the lentiviral vector. CMV (light grey arrow) indicates the promoter utilized to drive expression of the vector RNA in vector-producing cells. Transduction efficiency of the vector was analyzed by **(b)** florescence microscopy and **(c)** flow cytometry analysis in different cancerous B-cell lines. The scale of the microscope images is indicated by the red bar representing a length of 0.1 mm on all images.

To investigate the effects of miRNA expression on the Rituximab response in hard-to-transfect DLBCL cell lines, we first generated a lentiviral vector construct, pLV/miRCS-PE, with two expression cassettes allowing simultaneous expression of (i) a miRNA of interest expressed from the human U1 small nuclear RNA (snRNA) promoter and (ii) the eGFP reporter gene driven by a phosphoglycerate kinase (PGK) promoter (Fig 1a). Using expression of eGFP as a marker for successful gene delivery, we determined the transduction rate of upconcentrated LV/miRCS-PE in a total of six cancerous B-cell lines of DLBCL origin. Two of these cell lines were refractory to transduction by VSV-G-pseudotyped lentiviral vectors, but the remaining cell lines showed robust levels of lentiviral transduction (S1a Fig). In addition, the viability of cells after transduction was close to 100% for all cell lines (S1b Fig). Based on this screening for cell lines that were to susceptible to lentiviral transduction, we focused on the two GCB-like cell lines OCI-Ly-7 and SU-DHL-5 and the two ABC-like cell lines RIVA and NU-DHL-1. As determined by flow cytometry analysis, these four B-cell lines were all positive for CD20 expression on the cell surface (S2 Fig). For these cell lines, we performed additional transduction experiments and confirmed effective gene delivery by fluorescence microscopy (Fig 1b). Hence, levels of transduction determined by flow cytometry ranged from 57 ± 3% in RIVA cells to 78 ± 2.5% in SU-DHL-5 cells (representative expression profiles shown in Fig 1c). It should also be noted that the morphology of these four cell lines differed in a manner that was independent of lentiviral treatment. Hence, both OCI-Ly-7 and SU-DHL-5 cells formed clusters of cells with larger cell aggregates, especially evident in OCI-Ly-7 cultures, whereas neither RIVA nor NU-DHL-1 cells showed any tendency to cluster.

### Increased tolerance to Rituximab in GCB-like cells treated with lentiviral vectors

With the aim of exploiting lentiviral delivery in studies of Rituximab tolerance in B-cells, we set out first to identify cellular effects of lentiviral vector transduction. In a standard Drug Response Assay (DRA) (experimental setup depicted in S3 Fig), we exposed transduced cells to Rituximab in the presence of human serum (HS) 72 hours after transduction with LV/miRCS-PE and measured the effect of Rituximab treatment after additional 48 hours by counting the number of cells or analyzing labeling with BrdU as a measure of cell proliferation. Initially, OCI-Ly-7 and RIVA cells were treated with increasing doses of Rituximab, corresponding to GI50 (the drug concentration causing 50% inhibition of cell growth), TGI (the drug concentration causing total growth inhibition), and 2 × TGI.

**Fig 2:**
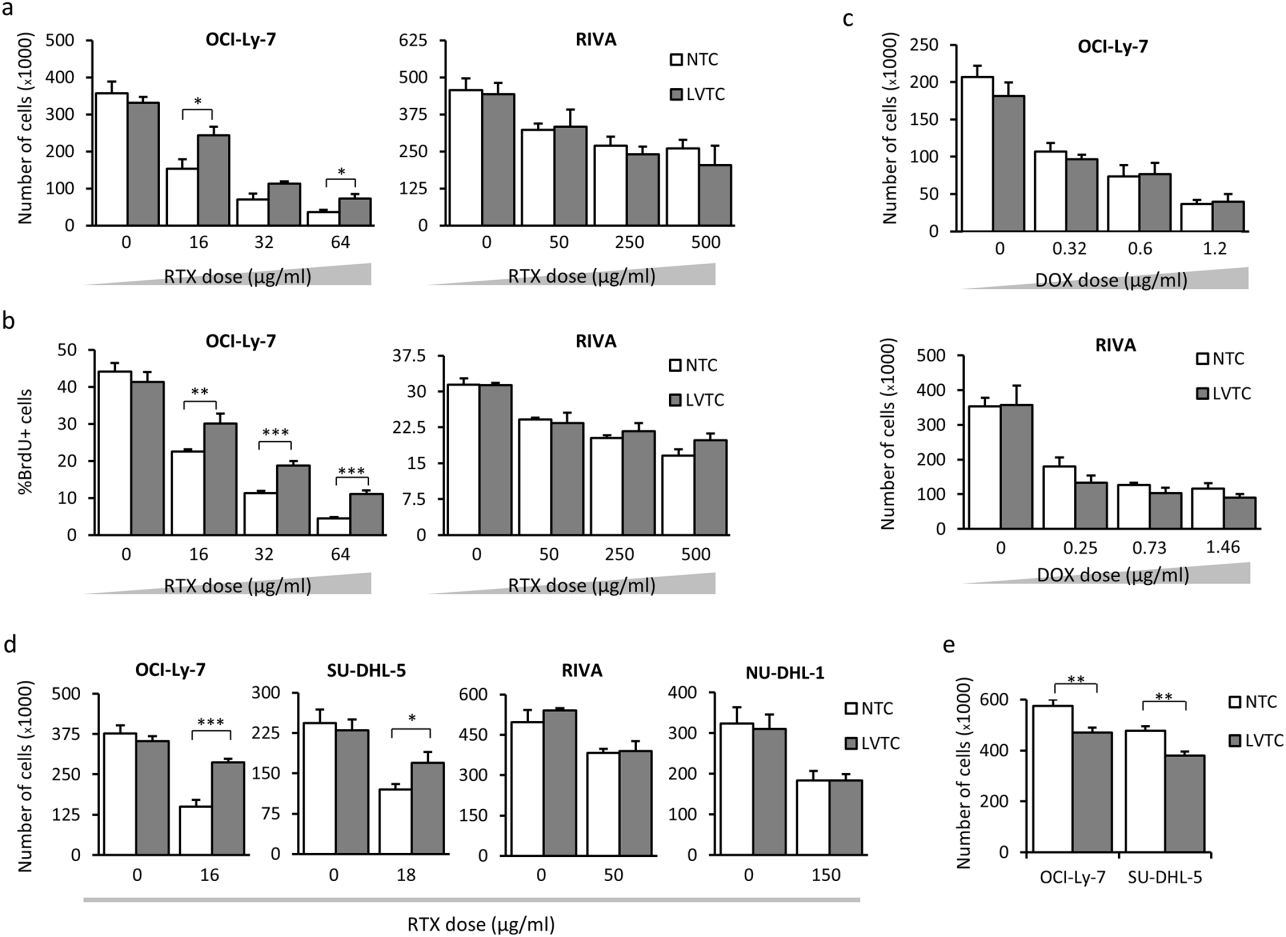
Lentiviral vector transduction increases Rituximab tolerance in GCB-Like cell lines. Cells were treated with Rituximab (RTX) 72 hours after lentiviral vector transduction. Cell proliferation was measured 48 hours after Rituximab treatment using **(a)** Trypan Blue staining and direct cell counting and **(b)** BrdU assay and flow cytometry analysis. **(c)** Lack of effect of Doxorubicin as shown by measuring the cell proliferation 48 hours after treatment with the drug. **(d)** Lentivirus-mediated increase of tolerance to Rituximab in GCB-Like DLBCL cell lines (OCI-Ly-7, SU-DHL-5), but not in ABC-Like cells. **(e)** Decrease of cell proliferation in OCI-Ly-7 and SU-DHL-5 cells treated with lentiviral vectors. Numbers of cells were determined after 96 hours of incubation. NTC, nontranduced cells; LVTC, lentiviral vector-transduced cells. Asterisks indicate level of significance as follows: *: *p* value≤0.05, **: *p* value≤0.01, ***: *p* value≤0.001.

For the OCI-Ly-7 cells we observed that cells transduced with the standard expression vector, LV/miRCS-PE, showed increased resistance to all doses of Rituximab (as determined by cell counting and BrdU proliferation assays) relative to cells that were not treated with a lentiviral vector (Fig 2a and 2b, left panels). Such improved tolerance could not be identified in transduced RIVA cells (Fig 2a and 2b, right panels). Treatment of the two cell types with Doxorubicin did not reveal any effects on the drug tolerance after lentiviral transduction (Fig 2c and S4a Fig), suggesting that the induced tolerance in OCI-Ly-7 cells was specific for Rituximab. Using a Rituximab concentration corresponding to the GI50, we performed Rituximab tolerance studies in all four cell lines (OCL-Ly-7, SU-DHL-5, RIVA, and NU-DHL-1 cells) and found the same effects of transduction with LV/miRCS-PE in the two GCB-like cell lines, whereas the tolerance to Rituximab was unaffected in the two ABC-like cell lines (Fig 2d and S4b Fig), suggesting that the induced tolerance was specific for GCB-like DLBCL cell lines. In parallel, direct cell counting of transduced OCL-Ly-7 and SU-DHL-5 cells showed a decrease in proliferation rate (Fig 2e), which was supported by analysis of BrdU incorporation (S4c Fig), illustrating that the Rituximab tolerance was not a result of increased cell proliferation in the lentivirally transduced cells.

### Lentiviral vector-induced Rituximab tolerance does not involve complement-mediated cell lysis

**Fig 3:**
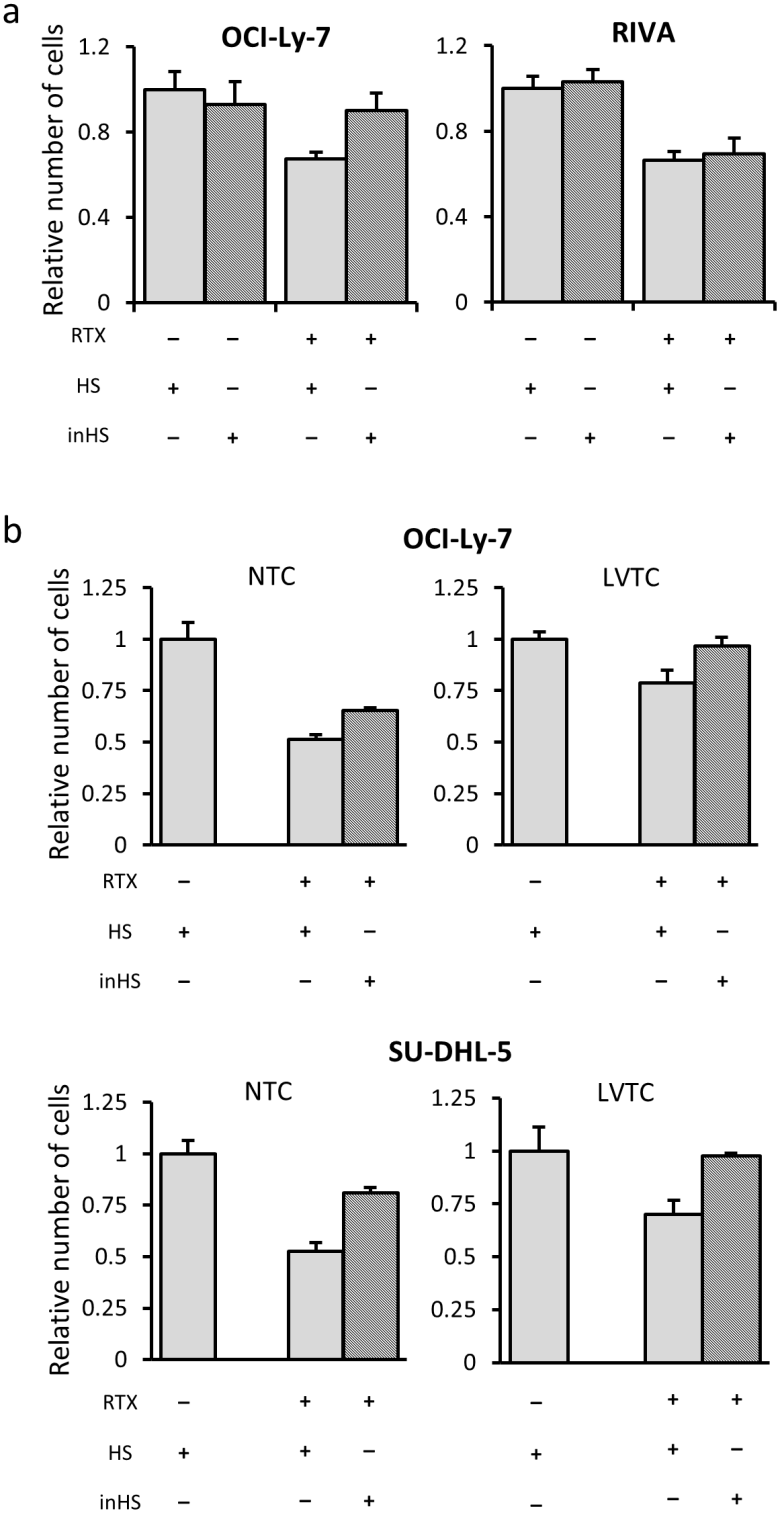
Induced Rituximab tolerance of GCB-like cells by a complement-independent effect of lentiviral vector transduction. **(a)** Increased tolerance to Rituximab (RTX) in GCB-like cells in the presence of complement relative to cells grown in the absence of complement. Treatment of cells with heat-inactivated human serum (inHS) showed that the apoptotic effect of Rituximab was independent of the complement system in RIVA (ABC-like) cells but not in OCI-Ly-7 (GCB-like) cells. **(b)** Relative survival rate of Rituximab-treated cells grown in human serum (HS) and inHS did not differ between nontransduced and lentivirally transduced cells. The number of OCI-Ly-7 and SU-DHL-5 cells was determined with and without lentiviral transduction in the absence or presence of active complement. For both GCB-like cell lines (OCI-Ly-7, SU-DHL-5) the relative increase in Rituximab tolerance was similar in NTC and LVTC groups, showing that protective effects of lentiviral transduction did not involve effects on CDC. Light gray and hatched columns represent cell numbers measured in the presence of HS and in the presence of inHS, respectively. The absolute number of cells was quantified by the trypan blue dye exclusion method after treatment with Rituximab and normalized to the number of untreated cells. The relative number of cells was used as a growth inhibition index showing the ratio of treated cells relative to the untreated control.

To investigate the role of complement-mediated cell lysis for the increased Rituximab tolerance in lentivirally transduced GCB-like cells, we analyzed the response to GI50 doses of Rituximab in the presence of HS and complement-inactivated human serum (inHS). We first cultured OCI-Ly-7 and RIVA cells, transduced with LV/miRCS-PE in the presence of inHS and HS. For OCI-Ly-7 cells, we found reduced tolerance to Rituximab in the presence of complement (cells grown with HS) relative to cells grown in the absence of complement (cells grown with inHS), as measured by counting cells (Fig 3a, left panel). In contrast, the response to Rituximab was not affected by complement in RIVA cells (Fig 3a, right panel). These findings were supported by measuring incorporation of BrdU (S5a Fig). Hence, cell death induced by Rituximab treatment of OCI-Ly-7 occurred partially through mechanisms involving complement dependent cytotoxicity (CDC).

We next set out to test whether the protective effect of lentiviral transduction in GCB-like cells involved effects on CDC. To this end, the number of OCI-Ly-7 and SU-DHL-5 cells was determined with and without lentiviral transduction in the absence or presence of active complement. For both cell lines, we observed increased tolerance to Rituximab of cells treated with lentiviral vectors independent of the presence of complement. Hence, the relative survival rate of Rituximab-treated cells grown in HS and inHS did not differ between nontransduced and lentivirally transduced cells (Fig 3b), which was confirmed by BrdU incorporation measurements (S5b Fig). This supports the notion that the protective effects of lentiviral transduction did not involve complement-mediated cell lysis.

### Lentiviral vector transduction induces anti-apoptotic effects in GCB-like cells

**Fig 4:**
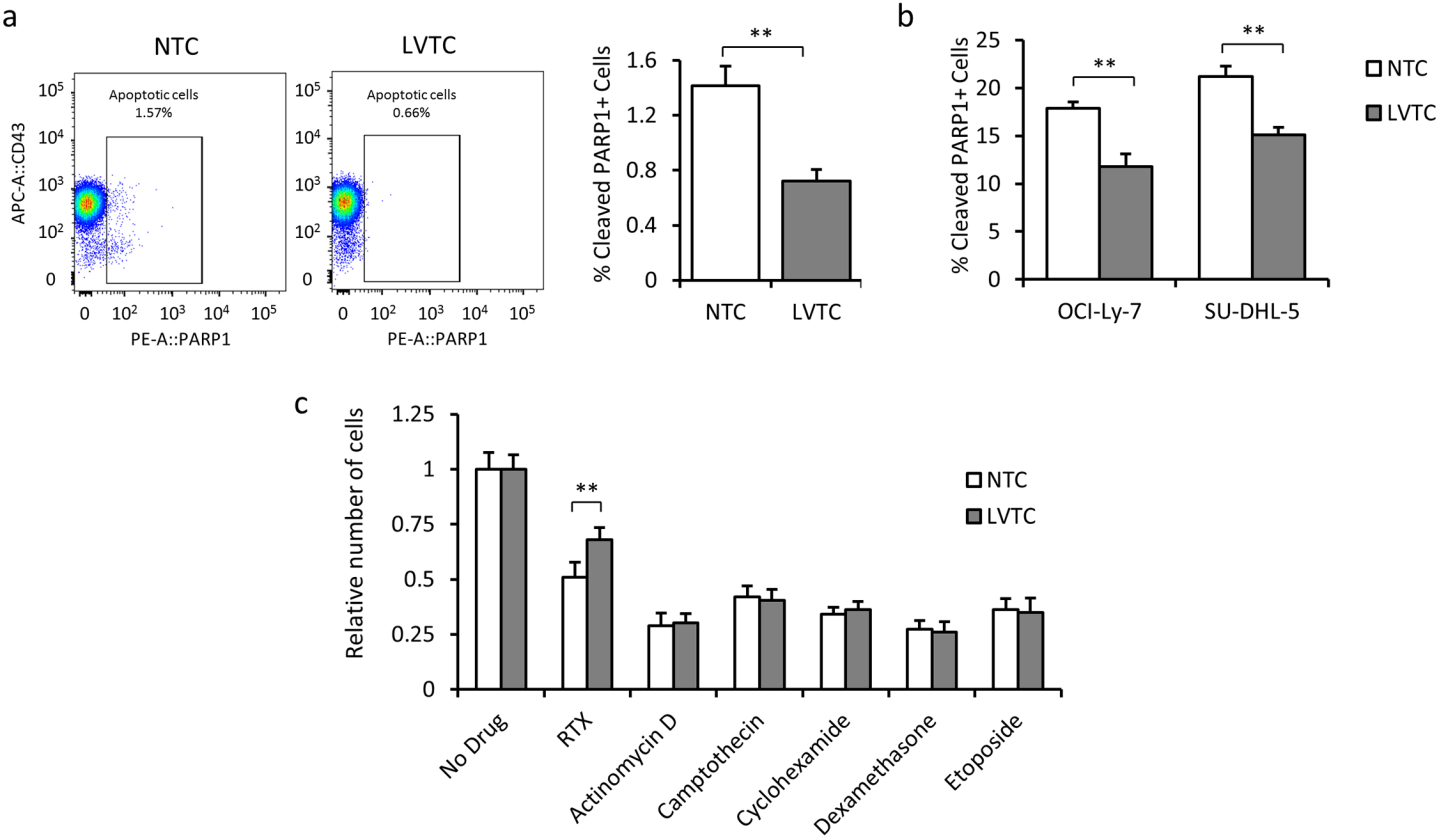
The anti-apoptotic effect of lentiviral transduction leads to acquired Rituximab tolerance. **(a)** Reduced apoptosis rate in lentivirally transduced versus non-transduced OCI-Ly-7 cell. **(b)** Reduced level of apoptosis in transduced cells relative to nontransduced cells exposed to Rituximab for 48 hours. **(c)** Analysis of potential anti-apoptotic capacities of lentiviral transduction in response to a series of apoptosis-inducing stimuli, including Rituximab (RTX) in OCI-Ly-7 cells. The relative number of cells was used as a growth inhibition index showing the ratio of treated cells relative to the untreated control.

Next, we addressed whether the protective effects of lentiviral transduction were associated with an altered apoptotic response. Using cleaved PARP1 as a measure of the apoptosis rate, we found that the number of cleaved PARP1-positive apoptotic cells in the absence of Rituximab and HS decreased from 1.4 ± 0.15% in nontransduced cells to 0.7 ± 0.1% in OCI-Ly-7 cells that were transduced with the lentiviral vector (Fig 4a). This anti-apoptotic effect of lentiviral transduction was significant also in OCI-Ly-7 and SU-DHL-5 cells treated with Rituximab (Fig 4b), demonstrating that cell death pathways were affected during lentiviral vector delivery. Notably, however, we tested the anti-apoptotic capacities of lentiviral transduction in response to a series of apoptosis-inducing stimuli (Actinomycin D, Camptothecin, Cyclohexamide, Dexamethasone, and Etoposide) and found increased cell numbers only in transduced OCI-Ly-7 exposed to Rituximab (Fig 4c), suggesting that the observed anti-apoptotic effects were specific for Rituximab. In summary, lentiviral transduction interferes with mechanisms of apoptosis that are specifically triggered by Rituximab.

### Persistent expression of CD43 in lentivirally transduced GCB-like cells treated with Rituximab

**Fig 5:**
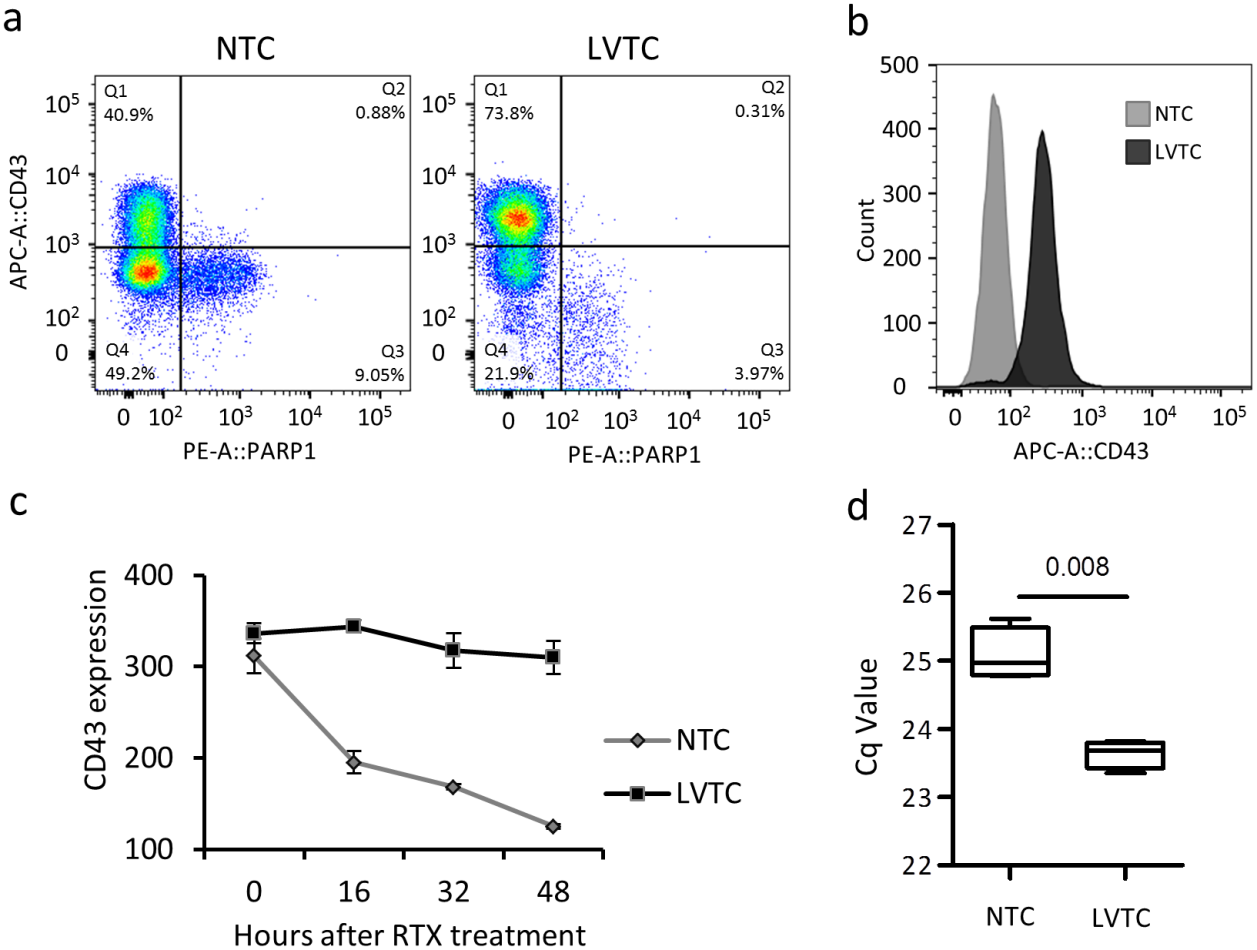
Loss of CD43 expression in response to Rituximab is blocked by lentiviral vector transduction through inhibition of apoptosis. **(a)** Reduced apoptosis in transduced OCI-Ly-7 cells 8 hours after Rituximab treatment, as measured by analysis of CD43 expression and cleaved PARP1 in NTC and LVTC. **(b)** Reduction of CD43 expression in OCI-Ly-7 cells 48 hours after Rituximab treatment. The histogram plot illustrates the number of CD43-positive cells in nontransduced cells (NTC), in lentivirally transduced cells (LVTC), and in a negative control cell line. **(c)** Loss of CD43 in response to Rituximab treatment is blocked by lentiviral transduction of OCI-Ly-7 cells. **(d)** Confirmation of maintained CD43 expression levels in cells transduced with lentiviral vectors and subsequently subjected to Rituximab. Analysis of CD43 gene expression was carried out using qPCR on total RNA isolated from NTC and LVTC.

Loss of expression of the surface protein CD43, expressed primarily in lymphocytes, is associated with initial stages of apoptosis in polymorphonuclear cells [40]. Reduction of the level of CD43 may therefore serve as a marker of cells undergoing early steps of apoptosis. In accordance, a population of OCI-Ly-7 cells subjected to Rituximab, showed increased levels of cleaved PARP1 in cells with lower levels of CD43, whereas cells with higher CD43 expression had lower levels of cleaved PARP1 (Fig 5a). Hence, cells with a lower CD43 expression profile showed higher level of apoptosis. Interestingly, we observed that the apoptosis (cleaved PARP1) rate for low-expressing CD43 cells (defined by the ratio between Q3 and Q4 in Fig 5a) was identical for the two groups (0.18 for both NTC and LVTC), demonstrating a constant level of apoptosis in cells with low expression of CD43. Cells transduced with lentiviral vectors were predominantly positive for CD43 and the rate of apoptosis, measured by cells with lost expression of CD43 or increased levels of cleaved PARP1, was markedly reduced (Fig 5a and 5b). We then followed the expression of CD43 in OCI-Ly-7 cells over time after Rituximab treatment and showed a gradual loss of CD43 expression in non-transduced cells. However, cells that were transduced with lentiviral vectors were able to resist the loss of CD43 expression, as shown by flow cytometry (Fig 5c) and confirmed at the RNA level by qPCR 48 hours after Rituximab treatment (Fig 5d). In conclusion, these findings suggest that anti-apoptotic effects contribute to increased tolerance to Rituximab of lentivirally transduced cells.

### Exploiting lentiviral transduction for analysis of the impact of miRNAs on Rituximab tolerance

**Fig 6:**
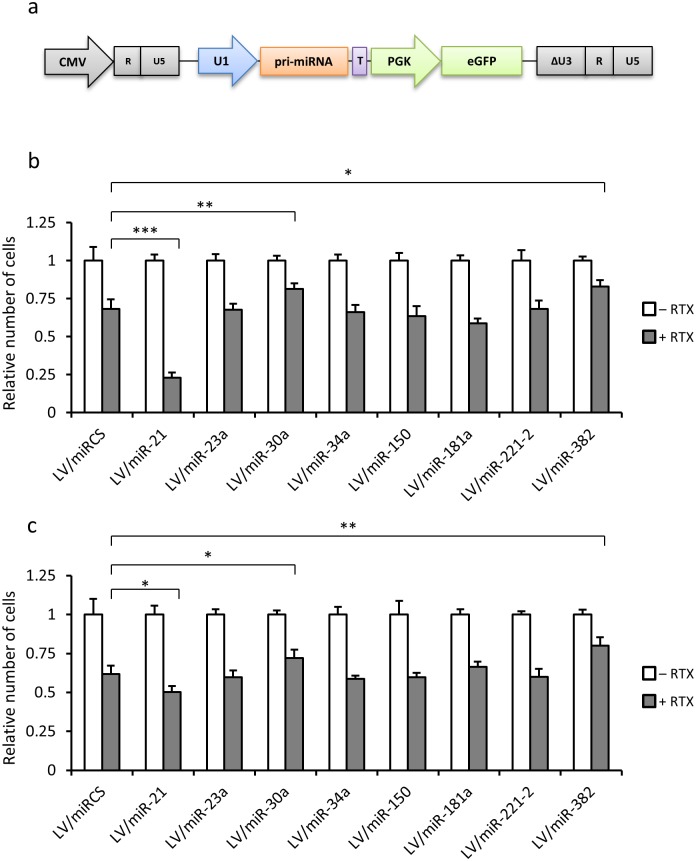
Exploiting lentiviral transduction for screening for miRNAs affecting Rituximab sensitivity. **(a)** Schematic representation of the lentiviral vector carrying a cloned pri-miRNA sequence derived from the human genome. Further details are provided in the legend to Fig 1. A panel of eight genomic sequences containing the miRNA sequence was inserted into vector, resulting in a set of eight lentiviral vectors. **(b)** and **(c)** Relative numbers of OCI-Ly-7 and SU-DHL-5 cells lentivirally transduced with vectors encoding eight different miRNAs. Cells were either grown in medium with Rituximab (+ RTX) or in medium without the drug and containing sodium chloride buffer as a control (–RTX). Cell numbers were determined by counting cells after staining with trypan blue. The relative number of cells was used as a growth inhibition index showing the ratio of treated cells relative to the untreated control. Statistical significance relative to the control group (cells treated with LV/miRCS-PE) is indicated by horizontal lines. Asterisks indicate level of significance as follows: *: *p* value≤ 0.05, **: *p* value≤0.01, ***: *p* value≤0.001.

Lentiviral vectors are unique tools for delivering genes effectively into B-cells and therefore also attractive, in studies of miRNA function. However, considering the effect of lentiviral transduction on cell death and the tolerance of GCB-like cells to Rituximab, it was unclear whether the effect of the delivery tool itself would interfere with studies of genes, including miRNA-encoding genes, affecting the tolerance to the drug. To address this issue in relation to the effect of specified miRNAs on Rituximab tolerance in GCB-like cells, we generated a series of eight lentiviral vectors, based on pLV/miRCS-PE, each expressing a pri-miRNA from a genomic sequence inserted downstream of the U1 promoter (Fig 6a). The miRNAs included in the analysis were selected based on their potential roles in DLBCL and earlier indications and clinical datasets pointing to a potential role of these miRNAs in modulating the response to Rituximab [28,41]. Details on each miRNA and the background for including this set of miRNAs in the analysis are provided in S6a Fig.

We first checked the functionality of the different miRNAs and in a dual targeting luciferase reporter assay confirmed that the entire panel of miRNAs, expressed from transfected plasmid DNA, was able to target and downregulate expression of a luciferase marker gene carrying the relevant miRNA recognition sequence (S6b Fig). Additionally, we analyzed transductional titers of all miRNA-encoding lentiviral vectors in both OCI-LY-7 and SU-DHL-5 cells and found robust gene transfer capacities resulting in 45 to 80% GFP-positive cells (S6c Fig). For each tested miRNA, we determined the cell count in the absence and presence of Rituximab and compared the relative cell number profile with the profile of control cells treated with LV/miRCS-PE, which did not express any miRNA. The screening of miRNAs was carried out in both OCI-Ly-7 and SU-DHL-5 cells, and comparable observations were made in the two cell lines. For a total of five miRNAs, miR-23a, miR-34a, miR-150, miR-181a, and miR-221-2, we were not able to detect any effect on the levels of Rituximab sensitivity (Fig 6b and 6c). However, in both cell lines expression of miR-21 induced increased cell death upon treatment with Rituximab, suggesting that this miRNA increased sensitivity to Rituximab. In addition, two of the tested miRNAs, miR-30a and miR-382, had the opposite effect upon lentiviral transfer, leading to a significant increase in the tolerance to Rituximab in both cell types (Fig 6b and 6c). These findings were confirmed for all three miRNAs by measuring BrdU incorporation (S7 Fig), although statistical significance was not seen for miR-30a. Together, our studies demonstrate the effective use of lentiviral vector-based miRNA delivery as a tool in studies focusing on the involvement of miRNAs in Rituximab sensitivity of GCB-like B-cells.

## Discussion

Lentiviral gene transfer is considered an effective tool for genetic intervention in hard-to-transfect cells, for example in relation to studies of miRNA function in different cellular pathways. The potency of lentiviral gene delivery is now also attracting more attention in functional studies and therapies focusing on B-cells [42–45]. However, the effects of lentiviral vector transduction in B-cells, and DLBCL cells in particular, remain unexplored, and the capacity of lentiviruses as a delivery tool in drug response studies is unclear. In the present study, we set out to investigate lentiviral transduction for delivery of miRNAs with a potential impact on drug responses in DLBCL cells and identified effects of lentiviral transduction on drug chemosensitivity. Despite such biological impact of incoming viruses, lentiviral vector transduction among a panel of miRNAs effectively identified specific miRNAs that impact the drug sensitivity in cancerous B-cells.

Our results show that lentiviral transduction with a control vector that does not express a miRNA interferes with Rituximab efficiency in GCB-like cell lines by increasing the drug tolerance. Different mechanisms of action have been proposed to explain the anti-tumor activity of Rituximab, including Antibody-Dependent Cell-mediated Cytotoxicity (ADCC), Complement-Dependent Cell-mediated Cytotoxicity (CDC), apoptosis induction, and cell growth arrest [8]. Hence, it is conceivable that one or more of these routes leading to B-cell death are affected during lentiviral gene delivery. Cellular changes that are reported to play roles in Rituximab tolerance include down-regulation of CD20, the antigen receptor for Rituximab [10–12]. In accordance, enrichment of regulatory B-cells expressing low levels of CD20 is a known cancer escape mechanism after Rituximab treatment [46]. Down-regulation of Bax and Bak, apoptosis-related proteins of the Bcl-2 family, is another mechanism of acquired Rituximab tolerance [13]. Moreover, inhibition of P38 MAPK activity has been reported as a potential mechanism for Rituximab tolerance [14]. However, in the context of our experimental set up based on cancerous cell lines we could narrow the effect of lentiviral transduction down to alterations of the drug response in relation to CDC and the induction of apoptosis and cell growth arrest. In accordance, we observed that Rituximab reduces the growth rate of GCB-like cell lines and ABC-like cell lines. For GCB-like cell lines this happens through mechanisms involving CDC and apoptosis, whereas ABC-like cell lines undergo cell growth arrest upon CDC (Maria Bach Laursen and Karen Dybkær, unpublished observations). It should be noted, however, that our platform in cultured B-cells does not support identification of potential effects involving an ADCC-based response. Although this may be looked at as a potential limitation of the cell model, studies in B-cell lines offer the advantage that the effect of Rituximab activity is limited to fewer cellular pathways. However, as ADCC may play an important role in Rituximab-mediated cell death *in vivo*, it is important to note that the lentiviral miRNA delivery platform may potentially allow studies of miRNA function in relation to ADCC responses in primary cells, including peripheral blood mononuclear cells (PBMCs). Notably, we have previously shown effective lentiviral miRNA delivery to PBMCs using vectors reminiscent of the vectors used in the present study [47]. Here, we used inactivated human serum to exclude complement-mediated actions of Rituximab in GCB cell lines and measured the apoptosis rate using cleaved PARP1 as a late apoptosis marker. We observed increased resistance to Rituximab in transduced cells relative to non-transduced cells, which confirmed that the acquired tolerance to Rituximab after lentiviral transduction was due to anti-apoptotic effects of the incoming lentiviral vector. This effect of transduction on apoptosis was not observed with Doxorubicin and a number of other apoptosis-inducers including Actinomycin D, Comptothecin, Cyclohexamide, Dexamethason, and Etoposide, supporting the notion that Rituximab induces apoptosis through a separate pathway that is affected by lentiviral vector transduction.

We also found that the level of CD43 expression after Rituximab treatment remained higher in transduced relative to non-transduced cells. CD43 is a large glycoprotein that can undergo several post-translational modifications to create different isoforms. It remains unclear, however, which roles these isoforms play in biological pathways. Some isoforms can improve cell survival [48], while others are necessary for cell death [49,50]. In the non-germinal center subgroup of DLBCL, CD43 leads to lower survival rate [51] and CD43-positive DLBCL patients respond poorly to Rituximab [52]. Also, CD43 has been reported to be a pre-apoptotic marker in polymorphonuclear cells (PMN), which decreases in early stage of apoptosis [40]. Our data demonstrate a negative correlation between apoptosis and the expression of CD43, which means that cells with lost CD43 expression have a higher risk of undergoing apoptosis. These observations may potentially suggest that CD43 can serve as a biomarker for the apoptotic response after treatment with Rituximab.

Our data showed that the lentiviral vector can efficiently transfer and express the miRNAs to cancerous B-cell lines. Screening among a panel of miRNAs for effects on the response to Rituxumab could be carried out using the standard vector as a negative control. Hence, Lentivirally mediated overexpression of miR-21 was found to dramatically increase the Rituximab effect in OCI-Ly-7 cells. This effect was less pronounced in SU-DHL-5 cells, which may correlate with the higher endogenous level of miR-21 in these cells. Furthermore, overexpression of either miR-30a or miR-382 augmented the resistance to Rituximab. MiR-382, in particular, demonstrated a protective effect against Rituximab. Although the molecular mechanisms for these effects remain a matter of speculation, these results together demonstrate the feasibility of exploiting lentiviral miRNA transfer for Rituximab sensitivity studies despite the acquired tolerance after transduction. Hence, considering that B-cells are difficult to transfect with plasmid DNA or synthetic RNA, lentiviral gene delivery remains a preferred method for carrying out genetic modifications in B-cells.

We have demonstrated that robust lentiviral transduction of cancerous B-cell lines markedly enhances the resistance of transduced GCB-like cells to Rituximab but not to Doxorubicin. This phenomenon involves anti-apoptotic effects that are specific for GCB-like cells and act independent of complement-directed cell lysis. Notably, we have found that cancerous B-cells lose the CD43 expression in response to Rituximab, and the effect of the drug is reduced in B-cells after lentiviral vector transduction. In summary, our results demonstrate that the biological impact of lentiviral transduction on cancerous B-cells may directly influence the outcome of drug sensitivity and point to CD43 as a potential marker for evaluating cancerous B-cell response to Rituximab. Thus, lentiviral vectors remain powerful tools for carrying out drug sensitivity studies related to the treatment of DLBCL.

## Supporting Information

S1 Fig Transduction efficiency and viability after transduction of different cancerous B cell lines. **(a)** Transduction efficiency of the pLV/miRCS-PE construct was measured by flow- cytometry in six different cancerous B cell lines. **(b)** Toxicity of the lentiviral treatment was checked in all cell lines using fixable viability staining and analysis by flow cytometry.
(TIF)

S2 Fig CD20 expression of cancerous B cell lines. Expression of CD20 on the surface of B cell lines OCI-Ly7, SU-DHL-5, RIVA, and NU-DHL-1 was measured by flow cytometry.
(TIF)

S3 Fig Experimental setup for Drug Response Assay. Flowcharts depict the experimental setup used to study Rituximab response in cancerous B cell lines after **(a)** lentiviral vector transduction and **(b)** miRNA overexpression.
(TIF)

S4 Fig Lentiviral vector transduction increases Rituximab tolerance in GCB-Like cell lines. Cells were treated with Rituximab (RTX) 72 hours after lentiviral vector transduction. BrdU incorporation was used to measure cell proliferation 48 hours after Rituximab treatment. **(a)** Lentiviral vector transduction did not change the Doxorubicin (DOX) response in OCI-Ly-7 and RIVA cells. **(b)** Lentivirus-mediated increase of tolerance to Rituximab in GCB-Like DLBCL cell lines, but not in ABC-Like cells. **(c)** Decrease of cell proliferation in OCI-LY-7 and SU-DHL-5 cells 3 days after lentiviral vector transduction. Asterisks indicate level of significance as follows: *: P value≤0.05, **: P value≤0.01.
(TIF)

S5 Fig Complement-independent induction of Rituximab tolerance in GCB-Like cells by a lentiviral vector transduction. Flow cytometry analysis of BrdU incorporation demonstrated **(a)** the independency of Rituximab (RTX) response to complement system in RIVA (ABC-Like) cells, but not in OCI-Ly-7 (GCB-Like) cells, and **(b)** the same level of relative survival rate in HS and inHS between lentivirally transduced and nontransduced GCB-Like cell lines (OCI-Ly-7, SU-DHL-5), indicating that lentiviral vector-mediated RTX tolerance is CDC independent. Light gray and hatched columns represent percentage of BrdU positive cells measured in the presence of HS and inHS, respectively.
(TIF)

S6 Fig Background information of selected miRNAs, functionality of cloned miRNAs, and transduction efficiency of miRNA-encoding LV/miR-PE variants. **(a)** Details on each miRNA and the background for including these miRNAs in the analysis. References are provided below. **(b)** Suppression of expression of the luciferase reporter gene carrying the miRNA recognition sequence by co-transfection with DNA plasmid vectors expressing relevant miRNAs. **(c)** Analysis of GFP expression 72 hours after transduction with LV/miR-PE vectors containing functionally verified miRNAs showed robust transduction in both OCI-Ly-7 and SU-DHL-5 cells.
(TIF)

S7 Fig Screening for miRNAs affecting Rituximab sensitivity. Cell proliferation was measured in **(a)** OCI-Ly-7 and **(b)** SU-DHL-5 cells by BrdU incorporation after lentiviral transduction with LV/miR-PE vectors encoding eight different miRNAs and LV/miRCS-PE as a control. Cells were either treated with the dose of Rituximab corresponding to GI50 (+ RTX) or subjected to the same volume of sodium chloride buffer (–RTX), and BrdU incorporation was determined by flow cytometry analysis.
(TIF)

S1 Table List of studied miRNAs and the primers used for PCR amplification. (TIF)
