# Supplementary figures and images for: Anti-Apoptotic Effects of Lentiviral Vector Transduction Promote Increased Rituximab Tolerance in Cancerous B-Cells
Source: PLoS One. 2016 Apr 5;11(4):e0153069. doi: 10.1371/journal.pone.0153069 (PMC4821607; doi:10.1371/journal.pone.0153069)

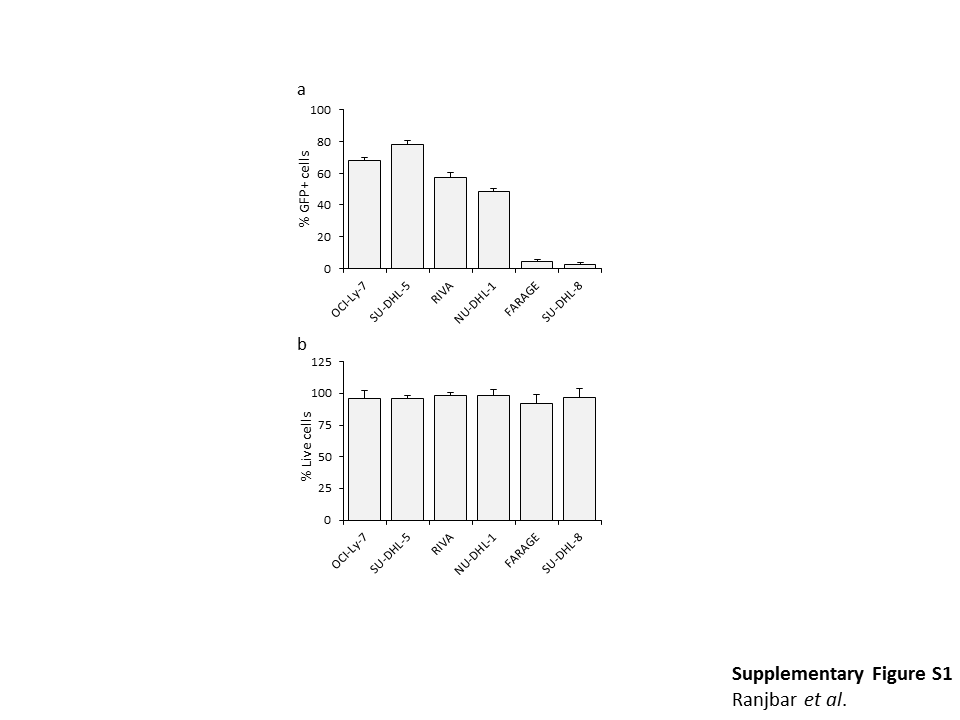

Supplement: S1 Fig — (a) Transduction efficiency of the pLV/miRCS-PE construct was measured by flow- cytometry in six different cancerous B cell lines. (b) Toxicity of the lentiviral treatment was checked in all cell lines using fixable viability staining and analysis by flow cytometry. (TIF) [file pone.0153069.s001.TIF]

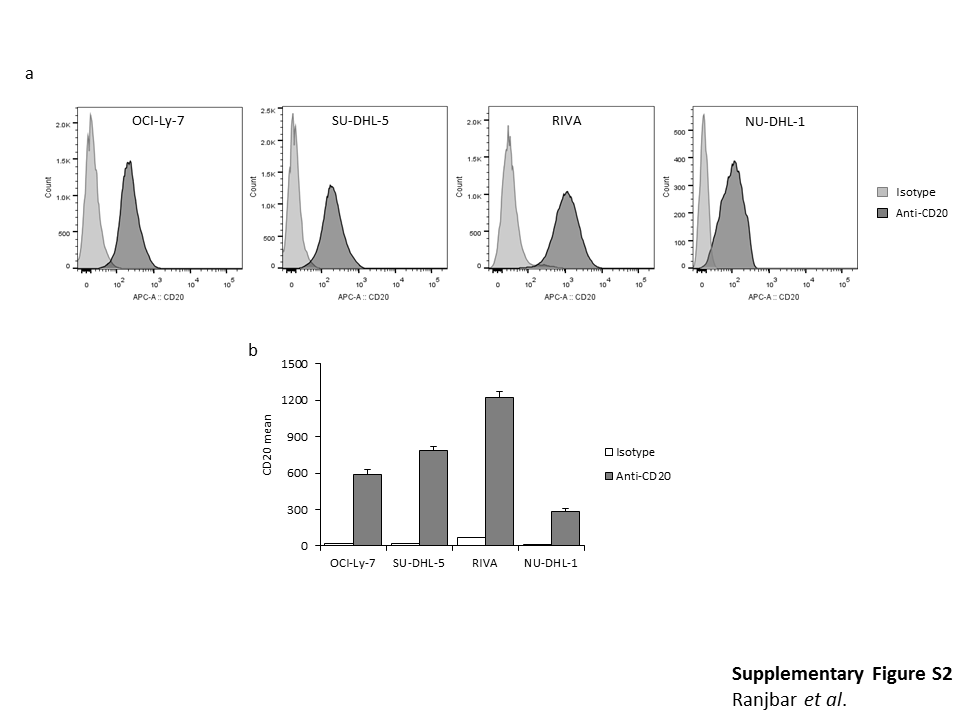

Supplement: S2 Fig — Expression of CD20 on the surface of B cell lines OCI-Ly7, SU-DHL-5, RIVA, and NU-DHL-1 was measured by flow cytometry. (TIF) [file pone.0153069.s002.TIF]

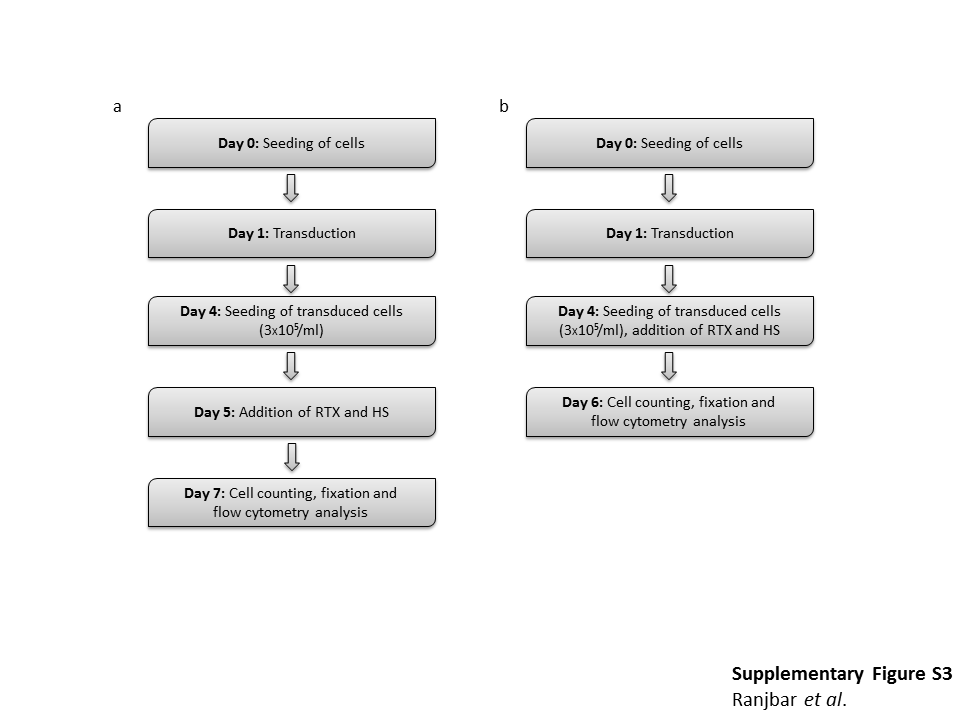

Supplement: S3 Fig — Flowcharts depict the experimental setup used to study Rituximab response in cancerous B cell lines after (a) lentiviral vector transduction and (b) miRNA overexpression. (TIF) [file pone.0153069.s003.TIF]

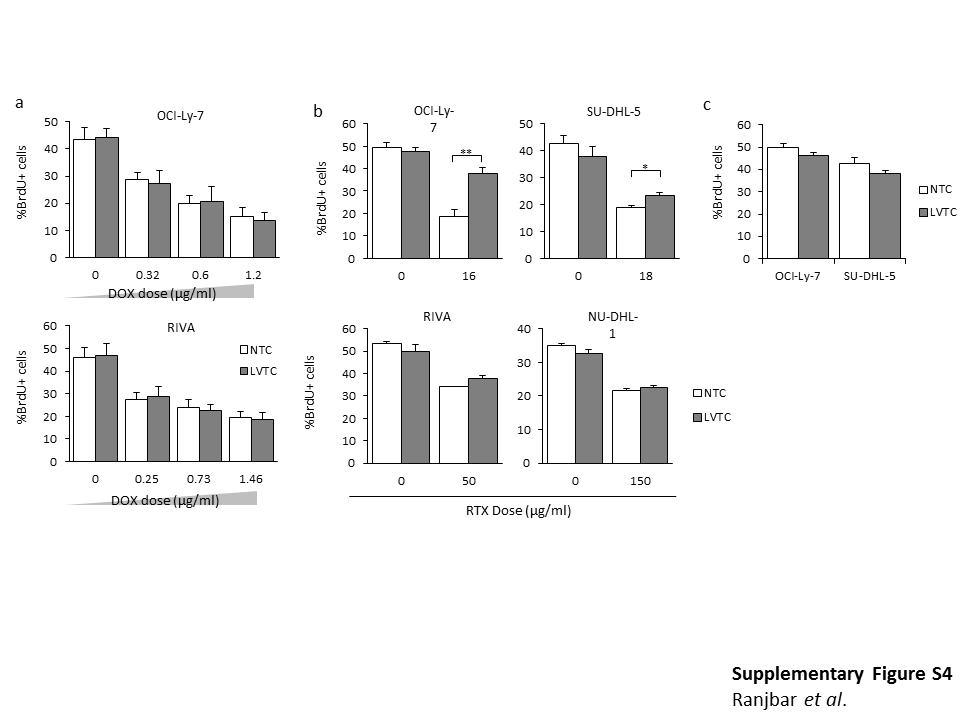

Supplement: S4 Fig — Cells were treated with Rituximab (RTX) 72 hours after lentiviral vector transduction. BrdU incorporation was used to measure cell proliferation 48 hours after Rituximab treatment. (a) Lentiviral vector transduction did not change the Doxorubicin (DOX) response in OCI-Ly-7 and RIVA cells. (b) Lentivirus-mediated increase of tolerance to Rituximab in GCB-Like DLBCL cell lines, but not in ABC-Like cells. (c) Decrease of cell proliferation in OCI-LY-7 and SU-DHL-5 cells 3 days after lentiviral vector transduction. Asterisks indicate level of significance as follows: *: P value≤0.05, **: P value≤0.01. (TIF) [file pone.0153069.s004.TIF]

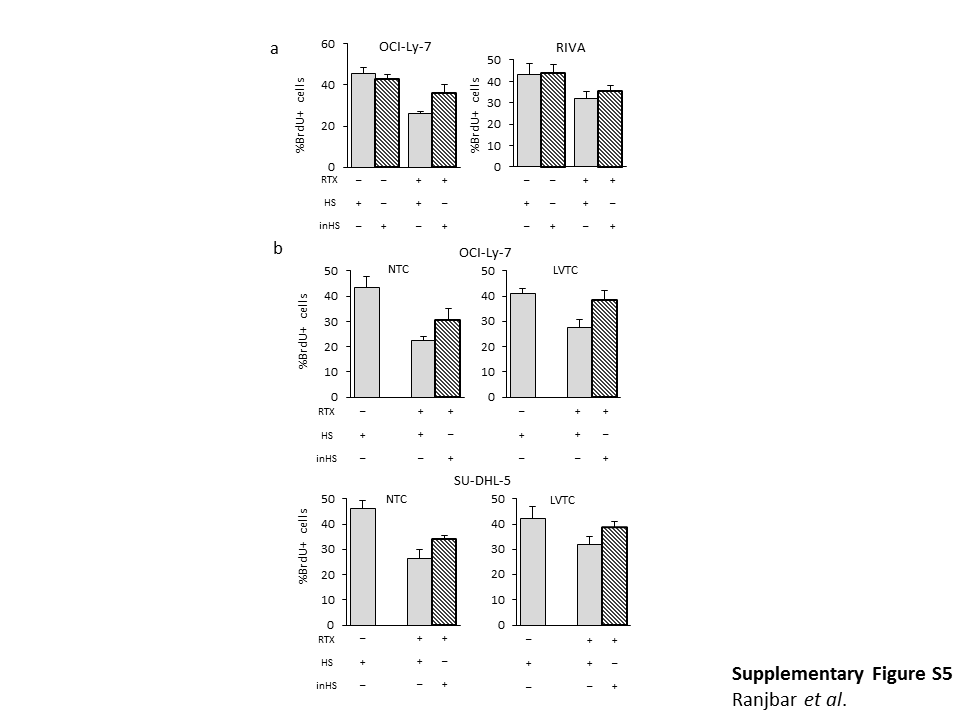

Supplement: S5 Fig — Flow cytometry analysis of BrdU incorporation demonstrated (a) the independency of Rituximab (RTX) response to complement system in RIVA (ABC-Like) cells, but not in OCI-Ly-7 (GCB-Like) cells, and (b) the same level of relative survival rate in HS and inHS between lentivirally transduced and nontransduced GCB-Like cell lines (OCI-Ly-7, SU-DHL-5), indicating that lentiviral vector-mediated RTX tolerance is CDC independent. Light gray and hatched columns represent percentage of BrdU positive cells measured in the presence of HS and inHS, respectively. (TIF) [file pone.0153069.s005.TIF]

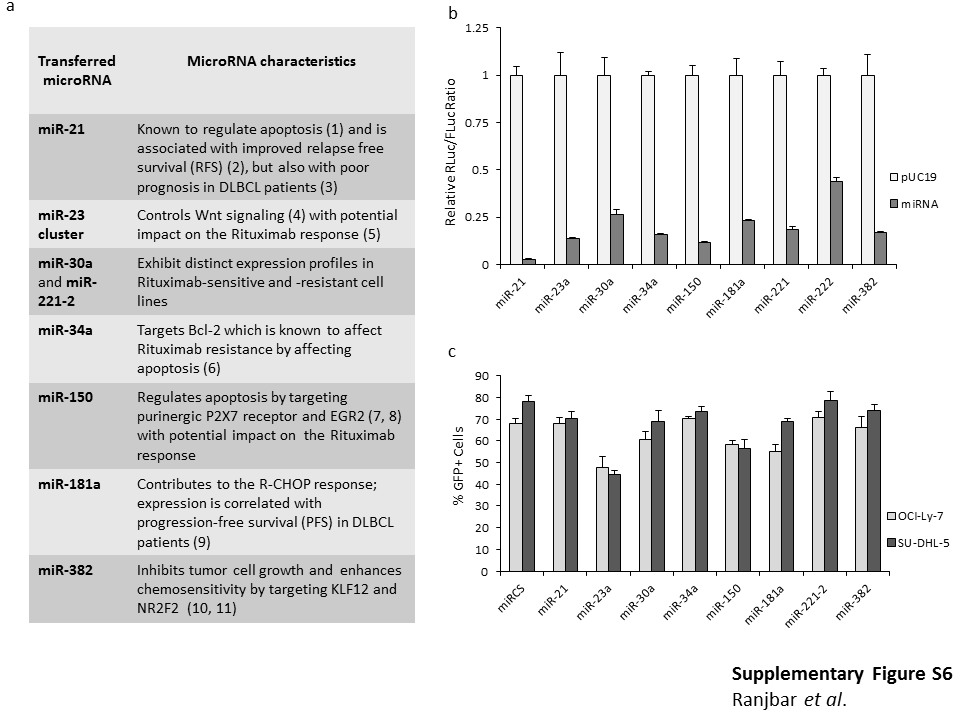

Supplement: S6 Fig — (a) Details on each miRNA and the background for including these miRNAs in the analysis. References are provided below. (b) Suppression of expression of the luciferase reporter gene carrying the miRNA recognition sequence by co-transfection with DNA plasmid vectors expressing relevant miRNAs. (c) Analysis of GFP expression 72 hours after transduction with LV/miR-PE vectors containing functionally verified miRNAs showed robust transduction in both OCI-Ly-7 and SU-DHL-5 cells. (TIF) [file pone.0153069.s006.TIF]

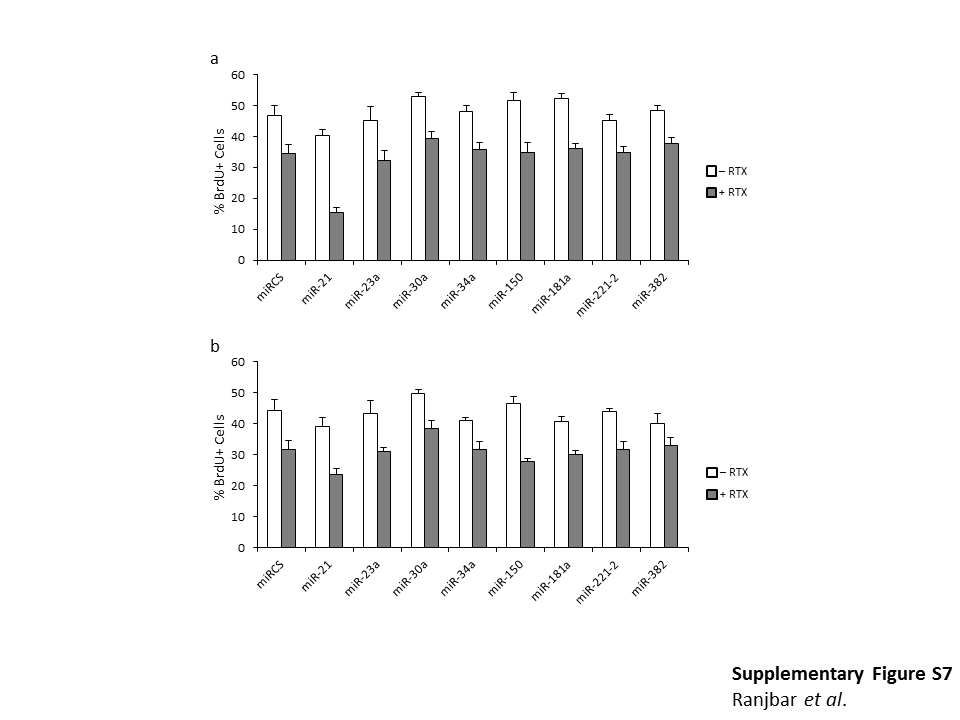

Supplement: S7 Fig — Cell proliferation was measured in (a) OCI-Ly-7 and (b) SU-DHL-5 cells by BrdU incorporation after lentiviral transduction with LV/miR-PE vectors encoding eight different miRNAs and LV/miRCS-PE as a control. Cells were either treated with the dose of Rituximab corresponding to GI50 (+ RTX) or subjected to the same volume of sodium chloride buffer (–RTX), and BrdU incorporation was determined by flow cytometry analysis. (TIF) [file pone.0153069.s007.TIF]

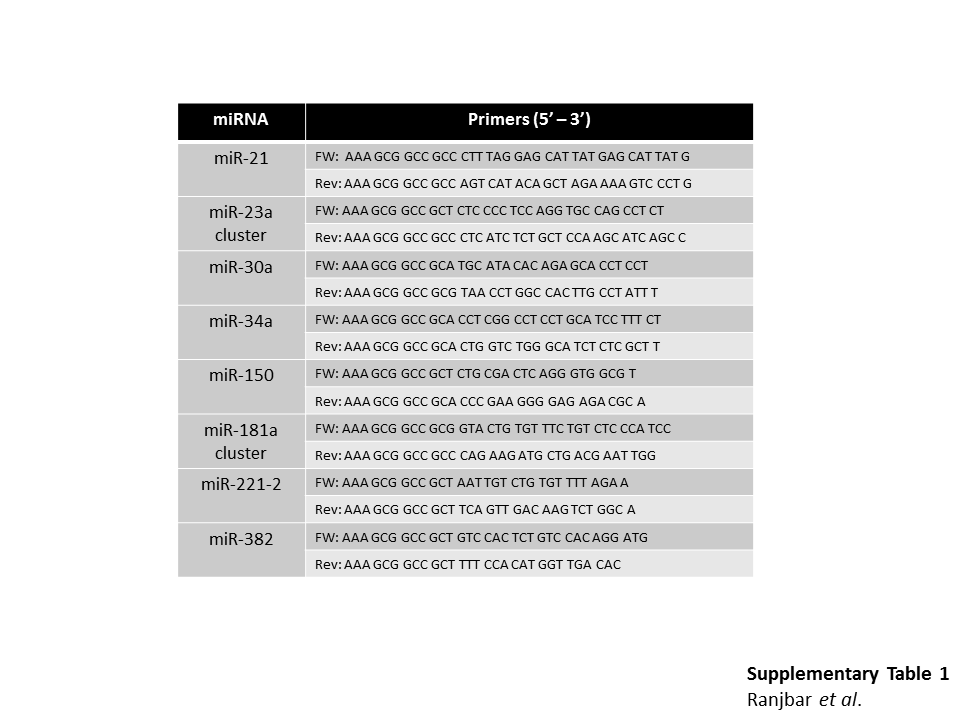

Supplement: S1 Table — (TIF) [file pone.0153069.s008.TIF]
